# An overview of the environmental pollution and health effects associated with waste landfilling and open dumping

**DOI:** 10.1007/s11356-022-21578-z

**Published:** 2022-07-01

**Authors:** Ayesha Siddiqua, John N. Hahladakis, Wadha Ahmed K A Al-Attiya

**Affiliations:** 1 Waste Management (FEWS) Program, Center for Sustainable Development, College of Arts and Science, Qatar University, P.O. Box 2713, Doha, Qatar; 2 Department of Environmental and Biological Sciences, College of Arts and Science, Qatar University, P.O. Box 2713, Doha, Qatar

**Keywords:** Waste landfilling, Solid waste, Environmental pollution, Health effects, Landfill, Waste management

## Abstract

Landfilling is one of the most common waste management methods employed in all countries alike, irrespective of their developmental status. The most commonly used types of landfills are (a) municipal solid waste landfill, (b) industrial waste landfill, and (c) hazardous waste landfill. There is, also, an emerging landfill type called “green waste landfill” that is, occasionally, being used. Most landfills, including those discussed in this review article, are controlled and engineered establishments, wherein the waste ought to abide with certain regulations regarding their quality and quantity. However, illegal and uncontrolled “landfills” (mostly known as open dumpsites) are, unfortunately, prevalent in many developing countries. Due to the widespread use of landfilling, even as of today, it is imperative to examine any environmental- and/or health-related issues that have emerged. The present study seeks to determine the environmental pollution and health effects associated with waste landfilling by adopting a desk review design. It is revealed that landfilling is associated with various environmental pollution problems, namely, (a) underground water pollution due to the leaching of organic, inorganic, and various other substances of concern (SoC) contained in the waste, (b) air pollution due to suspension of particles, (c) odor pollution from the deposition of municipal solid waste (MSW), and (d) even marine pollution from any potential run-offs. Furthermore, health impacts may occur through the pollution of the underground water and the emissions of gases, leading to carcinogenic and non-carcinogenic effects of the exposed population living in their vicinity.

**Graphical abstract:**

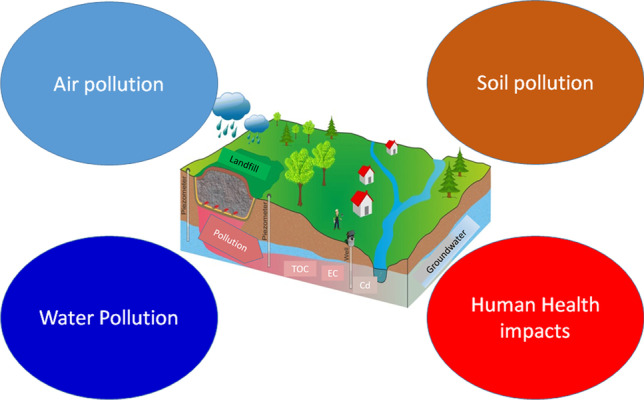

## Introduction

Environmental pollution has inherently been associated with health issues including the spread of diseases, i.e., typhoid and cholera, some of which are largely seen as waterborne diseases (Zhao et al. 2015). There are also non-communicable diseases (NCDs) that are brought about due to environmental pollution, such as cancer and asthma, or several defects evident at birth among infants (Reinhart and Townsend 2018). The significant adverse effects of environmental pollution on health-related outcomes have largely been evidenced in low-income countries, where an estimated 90% of the deaths are, in fact, caused by that type of pollution. The two most established forms of pollution in low-income countries are those of air and water. This is contrary to the economies that are rapidly developing, where the toxicity of chemicals and pesticides constitutes the main forms of environmental pollution (Xu et al. 2018).

Several human activities that include, among others, technological applications to change the ecosystems may, also, result in environmental pollution (Nadal et al. 2016). Other forms of pollution may be energy oriented, e.g., light, heat, sound, or several other chemical substances of concern (SoC). The pollutants can either be foreign energies/substances or contaminants that occur naturally (Gworek et al. 2016).

The urbanization and industrialization growth around the world has resulted into introduction of several SoC into the air, hence bringing about the respective type of pollution. It is through the earth’s atmosphere that life on our planet is fully supported (Duan et al. 2015).

Yang et al. (2018) identified five classes of pollutants: particulates, sulfur oxides, nitrogen oxides (NOx), hydrocarbons, and carbon monoxide (CO). In their study, they reported that in cities and centers, like Karachi and Islamabad, the leading air pollutants included carbon emissions and lead (Pb) (Yang et al. 2018). On the other hand, several types of water pollution exist, resulting in waterborne diseases (Joshi et al. 2016). Some of these waterborne diseases include typhoid, amoebiasis, and ascariasis. Various elements, depending on the concentration they occur, are considered toxic to humans. Therefore, if such an element is released in the air, water, or land, it can result into health complications/issues.

The different types of pollutants can be classified into inorganic, organic, or biological. Organic pollutants include the domestic, agricultural, and industrial waste that adversely harm the life and health of animals and human beings living on the earth. Inorganic pollutants mostly include the potentially toxic elements (PTEs), like mercury (Hg), lead (Pb), and cadmium (Cd). Most of these SoC get accumulated within supply chains, thereby largely harming the earth living organisms (Majolagbe et al. 2017). There are, also, biological pollutants that are anthropogenic derived. The key types of biological pollutants within the environment include viruses, bacteria, and/or several forms of pathogens (Marfe and Di Stefano 2016).

PTEs are regarded as one of the most important environmental pollutants, mainly due to their non-degradability, high persistence, and toxicity (Hahladakis et al. 2013, 2016). In their simplest form, PTEs occur naturally, and they have high atomic weight and density as compared to the one that water has. Of all the pollutants, greater attention has been given to PTEs (Mazza et al. 2015). Usually, these PTEs are present in trace levels in the naturally produced water, but the key challenge is that some of these PTEs are equally toxic even at low concentration levels. Some of these metals like zinc (Zn), cobalt (Co), Hg, Cd, and Pb and the metalloid arsenic (As) have high toxicity even when present in traces. When the body metabolizes these PTEs, they become toxic, being accumulated on soft tissues. There are various avenues through which these PTEs can gain access to human bodies, for instance, through absorption via the skin, food, and air, as well as water (Damigos et al. 2016).

There are various adverse environmental effects related with the PTEs. The majority of the PTEs are non-biodegradable and thus cannot go through degradation either chemically or microbially. Hence, their long-term influence is released via the ground and through the soil. At the same time, the PTEs can slowly find their way through drinking water which enters the human body. Reportedly, the contamination of water by PTEs has significant influence on all forms of animals (Annamalai 2015).

Toxic chemicals have emerged as a critical source of pollution all over the world. Their situation as environmental pollutants has largely been demonstrated and underpinned among low-income countries, where poor or inappropriate environmental controls take place. Common examples of toxic chemicals being major pollutants include any exposure to PTEs, e.g., Pb and Hg. Of the entire population across the planet, children are the most affected people when it comes to environmental pollution since any particle getting through their system may potentially results in long-term disabilities, as well as premature deaths (Kumar et al. 2017).

In an effort to prevent the aforementioned forms of environmental pollution, most countries have devised ways of preventing or minimizing any occurring impacts through proper disposal and/or burying of waste. Two ways are the most commonly applied: open dumping and/or landfilling. A dump is considered as an opening on the ground that is used for burying trash (Gavrilescu et al. 2015). On the other hand, a landfill is seen as a structure properly designed and built into or on the top of the ground. It is through a landfill that the necessary isolation of waste from the surrounding occurs. A controlled landfill ensures that waste is buried in an engineered manner, isolated from the ground water, while mostly maintaining the waste in a dry form (Indelicato et al. 2017b).

The rationale for the increased use of landfills is the environmental protection and prevention of pollutants entering the soil and, in turn, the underground water. This is obtained via a two way procedure: (a) application of a clay liner to ensure waste does not leave the landfill (sanitary landfills) and (b) application of synthetic liners, including plastic, to ensure that the landfilled waste is separated from the land (municipal landfill) (Mmereki et al. 2016). Although landfilling is structured with the aim of reducing waste, it may affect the three types of media previously identified and usually polluted (land, air, and water). After the waste is disposed in landfills, they are compacted to fill the entire area before being buried (Joshi et al. 2017). The rationale for this is to ensure that it will not come into contact with the environment. It, also, ensures that the waste is kept as dry as possible, limiting its contact with air so that it does not easily rot. It has been estimated that about 55% of the waste generated in the USA in 2008 was landfilled (US EPA 2008). Due to its widespread use, it is important to examine environmental pollution and health issues related with the landfills that have emerged across the world presently (Domingo et al. 2015).

## Methodology

The present study will adopt a desk review methodology. Przydatek and Kanownik (2019) define desk study as the collection of information from available sources, and it is one of the low-cost techniques, compared to field work (Przydatek and Kanownik 2019). During desk review, the study scans the available body of literature, carries out an analysis of the secondary data in place, and establishes a reference list at the end of the information/data collected. This helps in ensuring that the produced document is well organized and presented in a manner that is easily accessible.

Various scientific databases have been searched for this purpose, such as ResearchGate, ScienceDirect, eNature, JSTOR, LiveScience, Google Scholar, and Scopus. Different terms have been used in the search field areas, like “Water landfilling” AND “Health impacts” OR “Uncontrolled filling” AND “environment” “Health impacts” OR “Opened dump sites” AND “Health” OR “Landfills” OR “Pollution” OR “Dumpsite” “Environmental issues” OR “Health issues” OR “Waste management.” The produced results were narrowed down to include the last 10 years of publication from 2010 to 2020 to have an updated and critical review. The selected articles included both research and review articles. Upon this selection, the final results were then scanned for relevance to the review by previewing the abstracts and the titles. The relevant articles were then downloaded and reviewed thoroughly.

In the present review article, the delivered information will be organized under the following themes and sections: the third section, “Waste landfilling”; the fourth section, “Waste landfilling and environmental pollution”; and the fifth section, “Waste landfilling and human health risks.”

## Waste landfilling

**Fig. 1:**
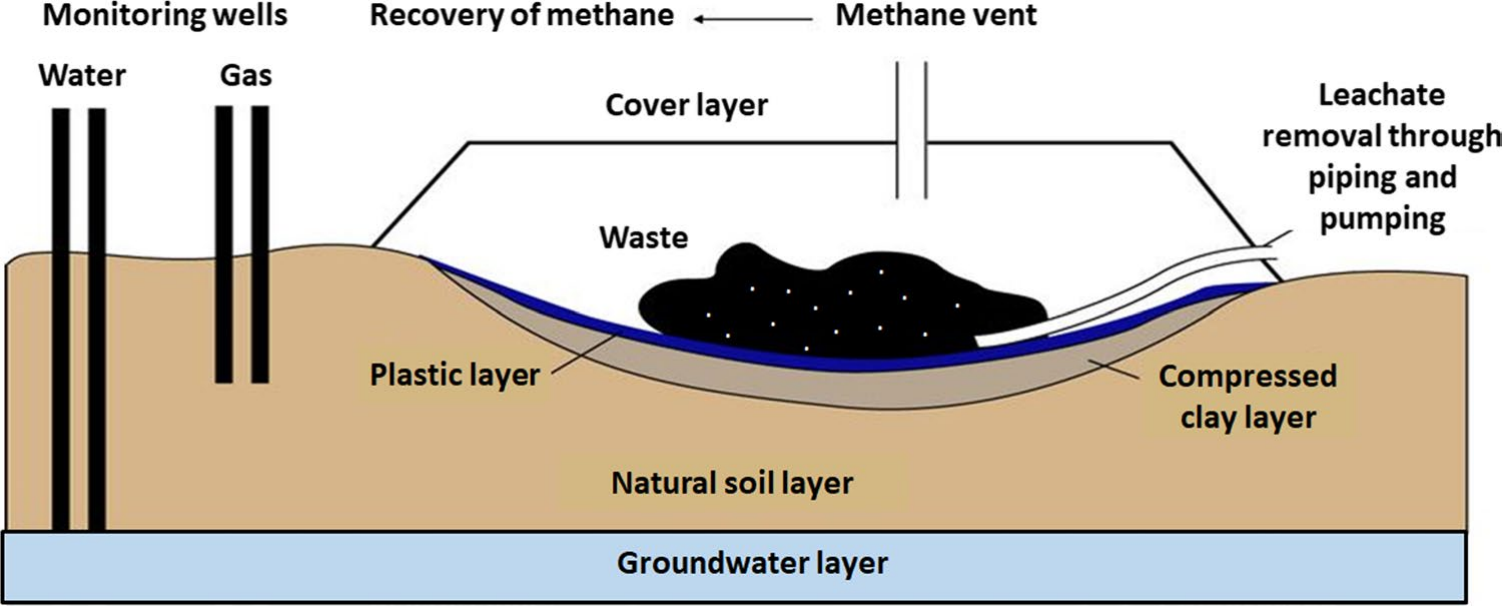
Typical layout of a waste landfill. (Redrawn from source: available at http://ocw.jhsph.edu)

A landfill is an engineered pit, particularly designed for receiving compacted solid waste and equipped with specific covering, so that the waste can be disposed of. There is a lining at the bottom of the landfill so to ensure that the waste does not pollute underground water (see Fig. 1). The design of landfills is such that they accept concentrated wastes in compacted layers so as to lower the volume.

The bottom of a landfill is protected to ensure that underground water is not contaminated. In essence, the deposited waste should be covered by soil at the end of each day. This will ensure that animals and flies are not able to dig up the waste. It also prevents undesired odors to get in the air and pollute the environment. In advanced — engineered — landfills, the bottom comprises of liner systems on the sides; there is also a leachate system and an underground monitoring system, as well as a gas extraction system. The gas extracted from landfills is used for energy production. There are, also, landfills possessing anaerobic or aerobic bioreactors: these help in accelerating the process of decomposition of organic waste within the landfill. The overall system provides, also, a conducive environment for microorganisms to decompose the existing waste.

The construction of landfills nearby residential areas is usually associated with effects like the accumulation of CH_4_ gases and contamination of underground water, as well as destruction of properties. This is particularly evident when landfills are not well engineered and/or maintained in a decent operational state; in such cases, there might be some leakages within the underground water, adversely affecting the life of the adjacent residents. In such a situation, people might need to consider relocating. In rural areas, most of the landfills are closed and small in size that rarely affect the quality of living; however, there might influence the value of the nearby properties.

### Types of waste landfills

The most commonly used types of landfills are (a) municipal solid waste landfills, (b) industrial waste landfills, and (c) hazardous waste landfills. There is, also, an emerging landfill type called “green waste landfill” that is, occasionally, being used. All the aforementioned types should, above all, be sanitary. So, before analyzing each independent type separately, it is considered necessary to elaborate and describe the “sanitary” term and present the main characteristics of a sanitary landfill.

#### Sanitary landfills

A sanitary landfill is simply a pit whose bottom is protected with a lining so that waste and other forms of trash are buried in layers, thus making it more solid/stable. It is at the sanitary landfills that waste is isolated from the environment in such a way that it is rendered safe. The waste is only considered to be safe after it has undergone complete biological, chemical, and physical degradation. The degree waste isolation within the sanitary landfills differs on the basis of the classification of the economies. For instance, in high-income economies, the degree of isolation is deemed to be very high (Ziraba et al. 2016).

The key role in the sanitary landfill is to ensure that all waste is placed in as safe as possible manner. It, also, facilitates safe decomposition of waste with the layers playing an important role in speeding up the process. The CH_4_ gas produced by the decomposition of the landfilled waste is harnessed and used to generate energy. Furthermore, the existing clay layer within the sanitary landfills ensures waste isolation from the environment (Rahmat et al. 2017). In addition, various designs and engineering methods are implemented since this is considered an important step in ensuring that there is no environmental contamination from the solid waste disposed in the sanitary landfills. In the event that the land used for the purpose of landfilling is filled up, impervious clay is used for sealing it and rendering it safe, so that the area can be further used for other activities (Qasim and Chiang 2017).

As earlier indicated, sanitary landfills largely operate by ensuring that waste is layered in large holes. There are various levels of layering that facilitate the entire process of waste decomposition, besides trapping the released toxic gases. The structure of these layers is such that the bottom part carries the smallest volume of waste, whereas the top part should bear the largest one. This is important to ensure that the surrounding land area does not collapse.

There are four specific layers within the sanitary landfills that play an important role in the entire process of the waste decomposition. The first layer is the one found at the bottom, which acts as the foundation of the sanitary landfill. This layer is made of dense and compact clay so that there is no waste seepage and thus no environmental (underground) pollution. It is on the basis of this reason that the clay used within the sanitary landfills is regarded as impervious (Rajaeifar et al. 2015).

The second layer is the drainage system. This layer protects the landfill from any decomposing that any waste oriented liquids could cause. Since this liquid is regarded as highly toxic, any seepage past the liner layer should be prevented. The role of the drainage system is to drain away the toxic liquids so that it does not get close to the liner system. At the same time, rainfall as well as snow may also create liquids that need to be drained out by this layer. Most of these liquids may contain contaminants that could result into corrosion of the liner system and/or contaminate the soil. In order to reduce these risks, the upper part of the landfills has perforated pipes on the greater part of the liner system. These pipes help to collect the liquids that may access the bottom of the landfills via leaching, hence the name leachates. This leachate is then directed to treatment plants via a plumbing system where it is treated for being reused (Adamcová et al. 2017).

The gas collection system constitutes the third layer of the sanitary landfills. Just as the way the liquids are produced within the landfills, gases are, also, naturally produced. One of these gases is CH_4_. CH_4_ is toxic, as well as volatile; thereby, its release to the atmosphere could significantly contribute to the global warming effect. To prevent this from happening, extraction pipes are used to ensure the CH_4_ gas is trapped and then transported to the plants for treatment and/or for generation of electricity.

Finally, the fourth layer is used to store the waste. This is the top and largest layer, used to store the waste collected by various companies. To minimize the space needed, the waste is compacted on a daily basis. At the end of this compaction process, a layer of compacted soil is applied on the surface of the sanitary landfill, so as to reduce any odors and the growth of microorganisms that are harmful, e.g., flies and pests.

Generally, sanitary landfills are designed to extend as deep as hundreds of feet, and it can take up to several years before being fully filled, after the compaction process. In the event that they are filled up, a capping is applied. In that case, a clay or plastic layer that is synthetic is introduced in the same manner as at the bottom. This is done to ensure that CH_4_ gas does not escape to the atmosphere and to prevent undesirable odors. At the same time, the top layers are firmly reinforced with an approximately 2–3 feet soil layer, and then plants are planted. In turn, this land may be reclaimed and used for other reasons.

However, despite all these safety processes and measures, there is a large possibility of underground contamination due to the high toxicity of the water oriented from the buried waste. The potential pathways of these toxic wastes may include the water, as well as cultivated soil for the production of edible plants. To minimize the risk, any filled or repurposed for gardening sanitary landfills are regularly monitored for decades. Their soil is, also, regularly tested to identify any irregularities. In the event any plants are dying, it could be an indication of CH_4_ release from the land. Only when the land has been tested and proven to be safe it can be used for other purposes. However, any heavy-duty activities, i.e., construction works, are not permitted in any case.

#### Municipal waste landfills

Municipal waste (also known as trash or garbage) is composed of all solid or semi-solid state waste and mostly includes domestic or household waste. The municipal landfills are one of the preferred methods for dealing with the largely increasing solid waste challenge. Municipal waste landfills are specifically designed so as to receive the household waste and other non-hazardous waste (Krčmar et al. 2018). As of 2009, there are approximately 1,908 municipal landfills in the USA, and these are managed by the states within the area of establishment (US EPA 2009).

#### Industrial waste landfills

An industrial waste landfill is where industrial waste is disposed of. While any type of solid industrial waste can be brought to these landfills, they are most often used for construction and demolition (C&D) waste disposal, which is why they are commonly known as C&D landfills. Waste could include concrete, gypsum, asphalt, bricks, and other building components (US EPA 2011).

#### Hazardous waste landfills

For obvious reasons, these types of landfills are the most closely regulated and structured landfills. They are specifically designed to hold hazardous wastes in a way that virtually eliminates the chance of it being leached and/or released into the environment. Some of the design requirements for hazardous waste landfills include double liners, double leachate collection and removal systems, leak detection systems, dispersal controls, construction quality assurance, etc. In addition to these design specifications, hazardous waste landfills undergo inspection multiple times a year to ensure that the facility is according to the latest high standards (Hazardous Waste Experts 2019; US EPA 2022).

#### Green waste landfills

While these landfills are not officially sanctioned landfills by the EPA, many municipalities are starting to adopt them for placing organic materials so as to get naturally decomposed. These composting sites are on the rise because most standard landfills and transfer stations are not accepting organic waste like fruits and vegetables.

Common types of green waste will include mulch, weeds, leaves, tree branches, flowers, biodegradable food waste, grass trimmings, etc.

The EPA has estimated that green waste landfills are making a bit of a difference with more than 24,000 tons of yard trimmings sent to these landfills in 2017 (US EPA 2017). The purpose of green waste landfills is to save space in other MSW landfills by keeping a material out that is meant to naturally decompose on its own.

### Theoretical underpinning

Various theories have been developed to explain the waste management and environmental conservation achieved through the establishment of landfills. These theories include the theory of environmentally responsible behavior (ERB), the reasoned/responsible action theory, the theory of planned behavior, the environmental citizenship, the model of human interaction with the environment and the value–belief–norm theory of environmentalism. The ERB theory was originally formulated by Hines, Hungerford, and Tomera in 1986 (Hines et al. 1986). The theory argues that having an intention to act is a key factor that influences responsible behavior for taking care of the environment. Moreover, it debates that the intention of acting, the locus of control, the attitudes, the sense of responsibility at the personal level, and knowledge are key tenets influencing the overall ERB (Akintunde 2017; Hines et al. 1986).

**Fig. 2:**
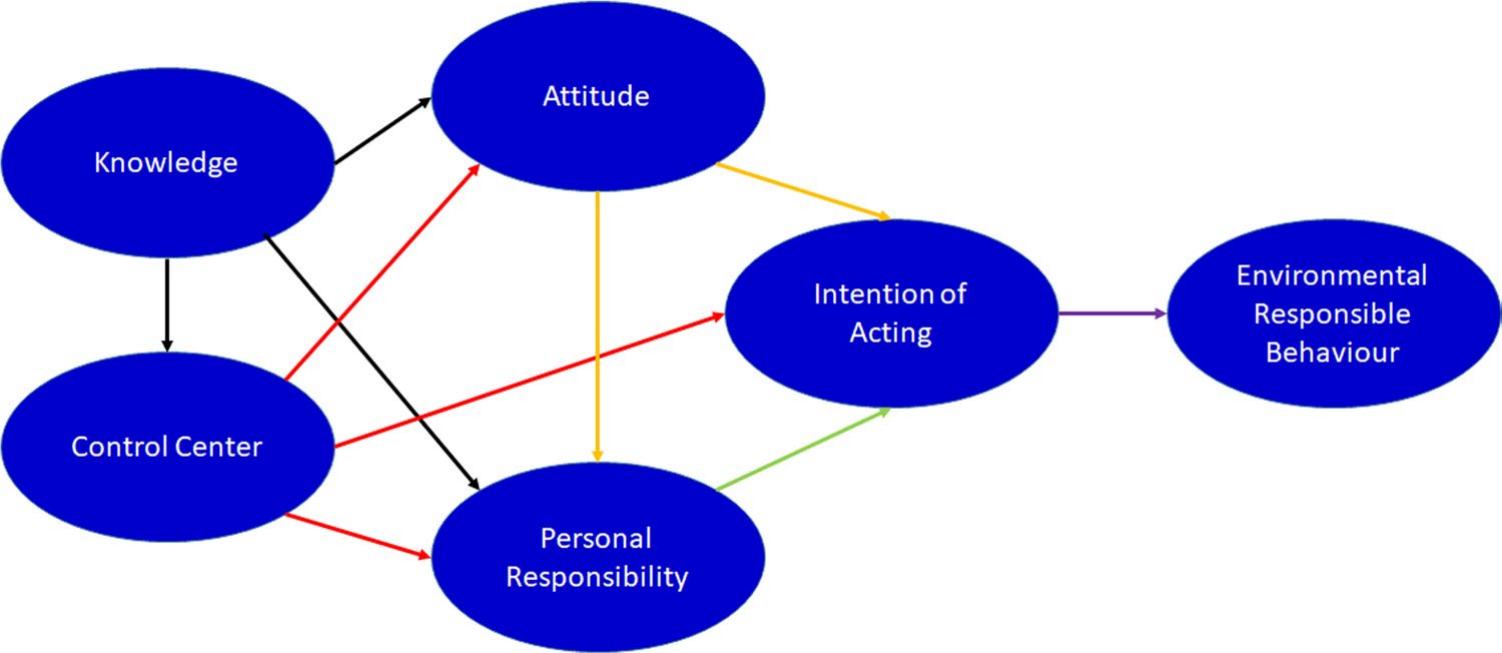
Schematic representation of the “Theory of Environmentally Responsible Behavior” (ERB). (Redrawn from source: Akintunde (2017)

The various interactions between the tenets of ERB are summarized in Fig. 2. According to this theory, the internal control center has an influence on the intention of people to act.

In the management of waste, no single factor exists that brings about a change in current behavior. For instance, despite the existence of stiff regulations forbidding people from damping waste materials, some people still damp waste or other materials in large cities. As indicated in Fig. 2, knowledge on its own is not adequate enough to lead to responsible actions and behaviors towards the environment.

**Fig. 3:**
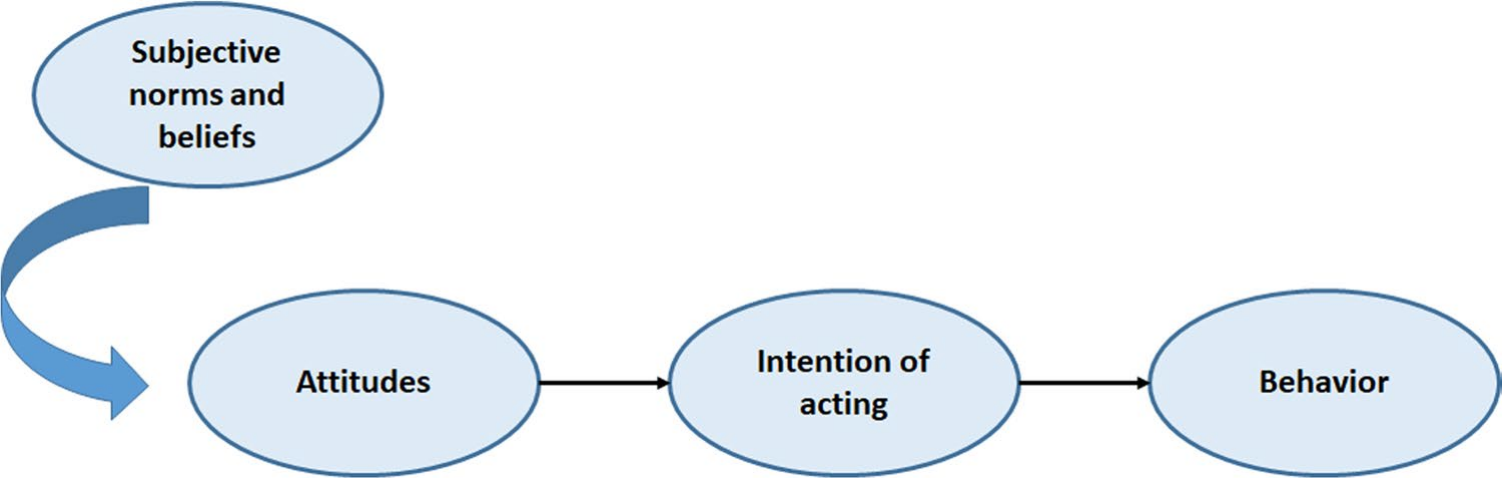
Theory of reasoned/responsible action. (Redrawn from source: Akintunde (2017))

The reasoned/responsible action theory was initially introduced by Martin Fishbein in 1967 and advanced and extended by Fishbein and Icek Ajzen (Akintunde 2017; Fishbein 1967). The theory argues that the various human behaviors are influenced and shaped by rational thoughts. According to this theory, there is a link between intentions to act and the final behavior of an individual as predicted by the attitudes. They are the subjective beliefs and norms that shape these attitudes. The theory of reasoned action is used to account for the time when individuals are guided by good intentions, but ensuring that these intentions are translated in good actions is affected by inadequate confidence Fig. 3.

## Waste landfilling and environmental pollution

Landfills have been regarded as the leading avenues that contribute towards emission of greenhouse gases (GHG) across the globe. This is because a large portion of gases, including carbon dioxide (CO_2_) and carbon IV oxide are released by the landfills to the air. It is the degradation process that results into all these gases polluting the environment (Papargyropoulou et al. 2015). In addition, the operations carried out at the landfills have been associated with contamination of the underground water sources through the produced landfill leachate. This occurs, particularly, when the liners within the landfills are not as adequate as required. There are, also, odors coming from the landfills that pollute the air, especially of those living in nearby areas. Other pollutants associated with landfills include dust, liter, and rodents (Ilankoon et al. 2018).

According to Hossain et al. (2014), landfill pollution is traditionally classified in several aspects. Maybe the most common categories are those that deal with the receiving air (emissions), water (effluents), and soil (dumps and disposals). A slightly more advanced breakdown would differentiate between inland and marine waters, surface and groundwater, and troposphere and stratosphere, and perhaps, considering the satellites and other types of debris, we should probably add outer space, as well. Most of the debate and regulation of pollution is based around these classifications, but focus is increasingly moving to inter-media impacts, such as the acidification of lakes and streams induced by air pollution or the disposal of sludge and other residuals from air and water pollution control measures on soil or in the ocean.

There are several factors that shape and determine the emission of landfill by-products: the quantity, as well as quality of deposited waste, the number of years a landfill has been operating for, and the climatic factors that surround it. There are some complicated microbiological and chemical reactions occurring within landfills that create gases to the air and hence air pollution. Some of the gases being released from landfills include sulfur dioxide (SO_2_) and as well as nitrogen dioxide (NO_2_), and these gases have an adverse effect on the environment. Inhaling any of these gases could result into throat and nose irritations that could potentially create asthma. Some of the landfill gases expose people that live around the area of such establishments with respiratory infections (Cucchiella et al. 2017).

**Fig. 4:**
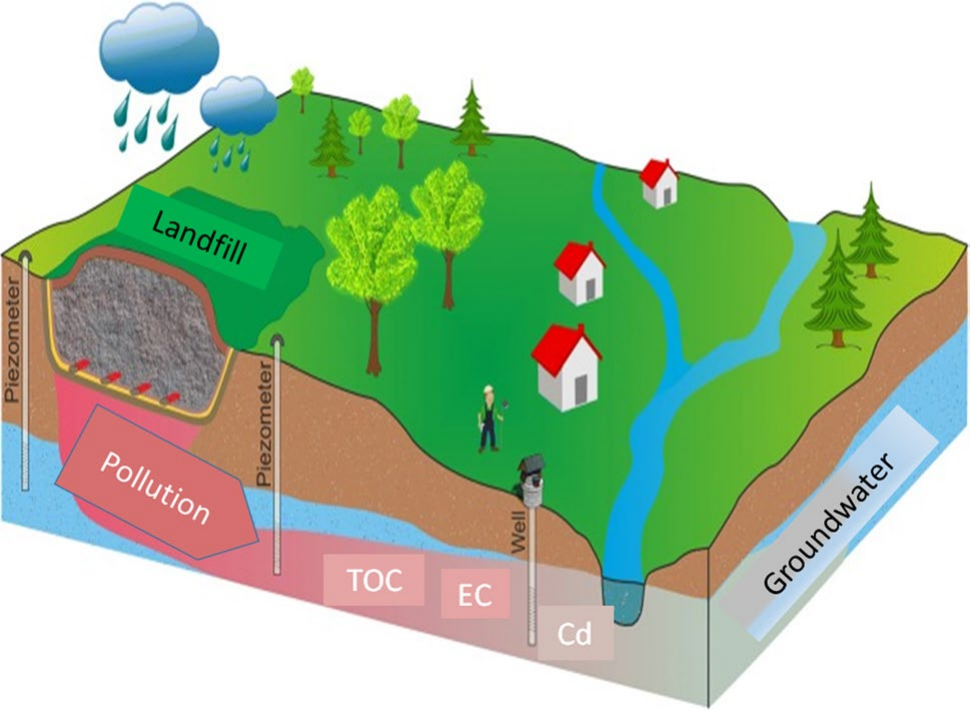
Route of underground water pollution-oriented landfills due to leaching. (Redrawn from source: SPREP (2010))

The rainfall on landfill sites results in dissolution of inorganic and organic elements of the landfilled waste. In turn, this releases toxic chemicals that leak to the underground water systems. Such type of water shall have high metal content, and it will be toxic if consumed by humans. In the event that these chemicals get towards the lake or river systems may pose adverse influence on aquatic life (Zhang et al. 2016). Waste landfills have, also, been associated with air pollution across the world. For instance, it is projected that about two-third of the landfills are made of organic materials that are biodegradable. The decomposition of these materials results into release of CH_4_ gas (Babayemi et al. 2016). This CH_4_ gas helps in trapping heat in the atmosphere since it is regarded as a GHG. The effect of waste landfilling on underground water pollution is illustrated in Fig. 4.

The development of waste landfilling affects, also, the biodiversity. For instance, developing the landfills implies that some 30–300 animal species are lost in every hectare. At the same time, there are some changes among the local species, where some of the birds and mammals are replaced with species feeding of refuse like crows and rats.

Njoku et al. (2019) performed a study in South Africa attempting to establish the link between landfills and environmental pollution. The formulated hypothesis was that the decomposed materials on landfills impact the environment of the surrounding area. It was shown from the results that about 78% of the people who live around these landfills are affected by air pollution. The people living close to landfills report, also, higher health issues including irritation of their eyes and flu. In this study, it was recommended to proper cover the landfill at the end of each day and place agents to dilute the odors (Njoku et al. 2019).

Vaverková et al. (2018) examined, also, landfills and their influence on the environment. In this study, it was shown that the investigated landfill had no direct and/or significant influence on the quality of water (Vaverková et al. 2018).

Danthurebandara et al. (2013) investigated the environmental impact of landfills and concluded that landfills do, actually, play a key role (Danthurebandara et al. 2013). However, it is from these landfills that approximately 20% of the global CH_4_ quantity is obtained. Besides CH_4_, there are gases released from these landfills that have high level of toxicity. It is possible that leachate can find its way through the underground water mainly via the flaws found on the liners. Constructing landfills may have an adverse influence in the life of fauna and flora.

Paul et al. (2019) reported in his study that municipal solid waste (MSW) treatment in Bangladesh had a large impact on the environment. More specifically, they reported that MSW leachate caused water pollution affecting, in turn, aquatic species. They, also, reported that open dumping caused soil pollution in Islamabad, affecting soil quality and thereby crop growth, production, and agriculture. Open dumping of solid waste in Nepal led to the spread of infectious diseases. They also reported that as landfills age, the process of mineralization of waste occurs which increases the leaching properties of the waste in the landfill (Paul et al. 2019).

Aljaradin and Persson (2012) studied the influence of landfills on the environment in Jordan. It was shown that the most widely used method for waste management is landfilling (Aljaradin and Persson 2012). However, it was reported that most of the landfills are associated with higher levels of pollution, with periodic leachate and the gas release to the underground water, creating an alarming environmental situation.

Mouhoun-Chouaki et al. (2019) conducted a study on landfills and their influence on the environment. Their specific focus was on establishing the influence of disposal of solid waste on the quality of soil within Nigerian landfills (Mouhoun-Chouaki et al. 2019).

Conte et al. (2018) examined the influence of landfills on air pollution with reference to Italy. It was found that landfills result to air, land, and water pollution to a large degree (Conte et al. 2018).

Adamcová et al. (2017) conducted a study on the environmental assessment of the effects of a municipal landfill on the content and distribution of PTEs in Tanacetum vulgare. Much attention was drawn to the effect of landfills on water sources, underpinning the need of taking mitigating actions since most of the population in the area depends on the water on a daily basis. It was, furthermore, reported that in terms of environmental contamination, social inclusion, and economic sustainability, landfill mismanagement is a worldwide problem that needs integrated assessment and holistic approaches/methods for its solution. Attention should be paid in developing and developed countries, where unsustainable solid waste management is prevalent. Differences should be identified between the development of large towns and rural regions where management problems differ, particularly with regard to the quantity of waste produced and the equipment available for landfill management (Adamcová et al. 2017).

Wijesekara et al. (2014) investigated the fate and transport of pollutants through a MSW landfill leachate in Sri Lanka. Due to the fast pace of natural resource exploitation, technological growth, and industrial expansion, the most striking reason for the landfill and thus worldwide environmental crisis is the deteriorating relationship between man and environment. The pace of change in the environment and its resulting degradation induced by human operations has been so rapid and common. Man’s effect on the environment through his financial operations is diverse and extremely complicated, as the natural situation and process transformation or alteration leads to a sequence of modifications in the biotic and abiotic components of the environment. Landfill mismanagement causes severe toxic metal pollution in water, soil, and crops, whereas open burning causes atmospheric pollutant emissions like CO_2_. Toxic metal-oriented environmental pollution is considered one of the most harmful types of contamination, particularly to human health. Finally, the authors of that study concluded that mismanagement of landfill is a serious danger to the environment as it inhibits sustainable development growth (Wijesekara et al. 2014).

Huda et al. (2017) investigated the treatment of raw landfill leachate via electrocoagulation and with the use of iron-based electrodes; all the parameters involved in the process were studied and optimized. Man’s environmental effects can either be direct and intentional or indirect and unintentional. Direct or deliberate effects of human activity are pre-planned and premeditated because man is conscious of the effects, both positive and negative, of any program initiated to alter or modify the natural environment for the economic development of the region involved. Within a brief period of time, the impacts of anthropogenic modifications in the setting are noticeable and reversible. On the other side, the indirect environmental effects of human operations are not premeditated and pre-planned, and these effects arise from those human operations aimed at accelerating the pace of economic growth, particularly industrial development. After a long time, when they become cumulative, the indirect effects are encountered (Huda et al. 2017). These indirect impacts of human economic activity can alter the general natural environment structure, and the chain impacts sometimes degrade the environment to such a degree that it becomes suicidal to humans.

Kalčíková et al. (2015) investigated the application of multiple toxicity tests in monitoring the landfill leachate treatment efficiency. Landfilling is still the prevalent option globally. It has been the main disposal technique of MSW in the latest decades as it is the easiest and most economical practice in many nations, especially in developing ones. Unfortunately, by hosting various stray animals and proliferating insect vectors of a lot of illnesses, these open landfills lead to severe health hazards. By producing both leachate and biogas, they also pose nuisance and significant environmental effects. The leachate conveys a significant pollution load that mainly consists of toxic metals, organic matter, and a significant community of pathogenic organisms: it causes organic, bacteriological, and toxic metal pollution of soil, surface water, and groundwater by leaching and ground infiltration.

Talalaj and Biedka (2016) conducted a study on the quality assessment of groundwater near landfill sites using the landfill water pollution index (LWPI). Due to the increase in human population and industrial and technological revolutions, waste management has become increasingly challenging and complicated, while processes that regulate the destiny of waste in the soil are complicated and some even poorly known. Sanitary landfill is the most popular and convenient technique of MSW disposal. Sanitary landfills provide better odor-free esthetic control. Often, however, unknown content industrial waste is mixed with domestic waste. Infiltration of groundwater and water supply contamination are prevalent. Unless properly managed, leaching and migration of SoC from waste sites or landfills and the release of various pollutants from sediments (under certain circumstances) pose a high threat to groundwater resources. Protection of groundwater has become a major environmental problem that needs to be addressed. Open dumps are the oldest and most popular way to dispose solid waste, and while thousands have been closed in the latest years, many are still being used (ISWA 2016). Some of the MSW disposal techniques that are frequently used include composting, sanitary landfilling, pyrolysis, recycling, and reuse (Talalaj and Biedka 2016).

Jayawardhana et al. (2016) investigated on MSW biochar for preventing pollution from landfill leachate. The immediate input of (primarily human) waste materials into the environment is usually connected with conventional or classic pollutants. Rapid urbanization and fast population growth have resulted in sewage issues as treatment facilities have failed to keep pace with the need. Untreated sewage from municipal wastewater systems and septic tanks in untreated fields contribute important amounts of nutrients, suspended solids, dissolved solids, petroleum, metals/metalloids (As, Hg, Cr, Pb, Fe, and Mn), and biodegradable organic carbon to the water ecosystem. Conventional pollutants can cause a multitude of issues with regard to water pollution. Excess suspended solids block the sun’s energy and thus influence the process of transformation of carbon dioxide–oxygen, which is essential for maintaining the biological food chain. In addition, elevated levels of suspended solids silt up waterways and channels of navigation, necessitating frequent dredging. For drinking and crop irrigation, excess dissolved solids render the water undesirable (Jayawardhana et al. 2016).

Another study conducted on an unlined MSW landfill in the Varanasi district of Uttar Pradesh in India showed that rainfall can have a major impact on the migration of leachate such as Fe, nitrate (NO_3_^−^,) total dissolved solids (TDS), phosphate (PO_4_^−^), and ions responsible for the electrical conductivity. Post monsoon, the groundwater quality, at several sampled stations, dropped either below the acceptable limit or the extent of groundwater pollution increased (Mishra et al. 2019).

The impact of landfill on the surrounding environment can be diverse depending on the different processes or methods that have been employed to it. In the work conducted by Yadav and Samadder (2018), different scenarios of MSW landfilling were studied, such as collection and transportation (S_1_); recycling, open burning, open dumping, and unsanitary landfilling without energy recovery (S_2_); composting and landfilling (S_3_); recycling, composting and landfilling (S_3_); and recycling, composting, and landfilling of inert waste without energy recovery (S_4_). It was found that each of the scenarios showed different degrees of environmental impact. For example, S_1_ had the highest contribution to ecotoxicity in the marine ecosystem; S_2_ contributed largely to global warming, acidification, eutrophication, and human toxicity; S_3_ had high impact on the depletion of abiotic resources such as fossil fuels and also responsible for aquatic and terrestrial ecotoxicity among others (Yadav and Samadder 2018). This demonstrates how a variety of processes can interplay in the landfill system to create a number of impacts, even with human interventions.

Although improper waste disposal results in the emissions of unwanted environmental pollutants such as GHG, a study conducted by Araújo et al. (2018) confirmed that simple sanitary landfills generated the highest amount of CO_2_, followed by sanitary landfill with CH_4_ collection, municipal incineration, and finally reutilization of woody waste (Araújo et al. 2018). This sheds some hope that proper intervention, such as reutilization and controlled release of pollutants, can be a potential method to reduce the emissions from landfilling.

Kazour et al. (2019) focused on the sources of microplastic pollution in the marine ecosystem. The study concluded that landfills close to the coastal waters were important sources of microplastic pollution in the ocean. Microplastics (MPs) were found in the leachate of active and closed landfills, suggesting that the location of the landfill also plays significant role in its characteristics of releasing plastics. The study found that inner lagoons with low water movement accumulated large amounts of MPs than the outer lagoon, which suggests that these MPs will be available as a contaminant in the marine environment (Kazour et al. 2019).

Another study conducted by He et al. (2019) reported that landfills that accumulate plastics do not act as final sinks for plastics but rather as a new source of MPs. They suggested that these MPs undergo breakdown due to exposure to the UV light and the prevalent conditions in the landfill (He et al. 2019). This study underpinned the impact of the landfill on coastal environments which are considered fragile ecosystems harboring large diversities.

Meanwhile, a study conducted by Brand and Spencer (2019) investigated the ecological impact of historical landfills located in the coastal zones. They reported that changing climate and proximity to coast can increase the changes of waste release into the waters due to erosion, storms, or even the collapse of the landfill due to age and infiltration of water. Historic landfills are unregulated as they predate modern environmental regulations and are no longer maintained or managed by previous operators. Thus, unmanaged landfills have detrimental impact especially because such landfills can have a wide mixture of waste. The authors of this study speculated that any metal release (derived from the wastes) to the adjacent Thames estuary, should they erode completely, will, i.e., increase the copper (Cu) levels 6.4 times. This will have long-term ecological impacts on the flora and fauna in the immediate vicinity and throughout the marine ecosystem. As of now, most metals exceed interim sediment quality guidelines (ISQG) levels (Brand and Spencer 2019). This study highlights the importance of maintaining the landfills of today’s society and their maintenance. Future considerations must also be made to existing landfills so that they may be managed well into the future without threatening the societal ecological balance.

Adamcová et al. (2017) pointed in two ominous directions: (a) towards big and increasing release of certain chemicals, primarily from burning fossil fuels, which are now considerably modifying natural systems on a worldwide scale, and (b) towards constant rises in the use and release of countless biocide goods and poisonous substances into the atmosphere. These raise a more severe issue presenting tremendous problems to the societies, both developed and developing. They concluded that several large-scale social and technological transitions are required to tackle the severe pollution problems in the coming decades (Adamcová et al. 2017).

Guerrero-Rodriguez et al. (2014) suggested that today’s pollution from landfill is integrally linked to financial manufacturing, contemporary technology, lifestyles, sizes of populations of humans and animals, and a host of other variables. Except for wide macro-transitions with various social benefits, it is unlikely to yield. These transitions include moving away from fossil fuels and waste-intensive techniques, bringing to bear our most advanced science, changing prices and other financial incentives, perceiving emissions as either trans boundary or global, and moving towards world population that is very stable (Guerrero-Rodriguez et al. 2014).

According to Majolagbe et al. (2017), land is frequently used as a waste treatment recipient, accepting spills of waste. Land pollution is the degradation of the earth’s land surface by bad farming methods, mineral exploitation, industrial waste dumping, and indiscriminate urban waste disposal. For a lot of municipal and some industrial waste, recycling of materials is practical to some extent, where a tiny, but increasing percentage of solid waste, is being recycled. However, when waste is mixed, recovery becomes hard and costly.

The former statement has been analyzed, along with new proposed methods in order to sort ferrous and nonferrous metals, plastics, paper, glass, etc., and many communities are implementing recycling programs that require separation of commingled waste. Developing better handling techniques, inventing new products for recycled materials, and finding new markets for them still remain crucial problems for the recycling sector (Hahladakis and Aljabri 2019; Hahladakis and Iacovidou 2018, 2019; Hahladakis et al. 2018; Majolagbe et al. 2017).

## Waste landfilling and human health risks

Love Canal is one of the most widely acknowledged landfill which is located in New York. During the periods of the 1930s to the 1940s, a huge volume of toxic materials was deposited. This was followed by establishing residential houses and learning institutions around this landfill in the 1950s. As of the mid-1970s, a number of chemicals were detected to have been leaked to the nearby streams and sewers. This has resulted into various studies being carried out to explore how this affected the human health. Most of the studies carried out have revealed that landfilling has, indeed, been associated with health issues, as a result of emissions of SoC to the air.

In Italy, studies have been carried out to reveal any effects associated with living closer to areas where there is landfilling. It was revealed that hydrogen sulfide (H_2_S) was associated with lung cancer and other respiratory health issues. The most affected part of the population was the children.

Vrijheid (2000) reported on the health issues that are related with people living closer to landfilling. The trigger point for this study was the fact that some specific form of cancer and defects at birth as well as low birth weight have been linked with individuals that live closer to landfilling areas. It was shown that living closer to landfilling areas is associated with respiratory diseases like asthma. This is largely attributed to the emissions of the gases to the air that affect the health outcomes of individuals (Vrijheid 2000).

Limoli et al. (2019) reported that illegal landfilling has adverse health effects on people living near the landfills and that it is more harmful to children, as their immune systems are still developing and because they spend most of the time outside their homes. They noted that health impacts can range from acute intoxication to carcinogenicity, endocrine-related toxicity, genotoxicity, and mutagenicity, depending on the contaminants. Upon contact with water, some contaminants dissolve and leach into the soil and contaminate the underwater table. Such pollutants that dissolve into the liquid phase include ammonium nitrogen that can cause eutrophication, chlorides that can alter the reproductive rates of marine animals and plants, organic matter that contributes to the deterioration of the water quality, persistent organic pollutants (POPs) that can cause bioaccumulation, and biomagnification in the food chain and sulfates that may increase nutrient levels in the water body, leading to eutrophication, in addition to fostering the production of methylmercury by some bacteria which is toxic. As part of the gaseous emissions, NOx triggers photochemical smog and contributes to acid rain and phytotoxic, particulate organic matter reduces photosynthetic rate and aids in photochemical smog formation, sulfur oxides cause acid rains, and volatile organic compounds (VOCs) cause the formation of harmful ground-level ozone. Besides these, many types of hazardous wastes can also be added such as PTEs that lower water quality; radionuclides and pathogenic waste are severely harmful for the living organisms (Limoli et al. 2019).

Mattiello et al. (2013) sought to determine how disposing solid waste in landfills affects health outcomes. The study systematically reviewed the available information on the subject under consideration. It was shown that the health issues linked with landfills include respiratory diseases and possible hospitalization especially among children (Mattiello et al. 2013). Maheshwari et al. (2015) focused on landfill waste and its influence on health outcomes. The review of information showed that landfills are associated with air, water, and land pollution problems around the world. These forms of pollution have adverse influence on people especially children who have weak immunity systems. Pollution of the environment through dumping of waste is associated with health issues on a long-term basis. The gases that are emitted from the landfills result into environmental pollution, and they are also associated with a number of issues related with cancer (Maheshwari et al. 2015).

Xu et al. (2018) conducted a study to find out the correlation of air pollutants associated with land filling on the respiratory health of children living in the proximity of a particular landfill in china. They reported that CH_4_, H_2_S, CO_2_, NH_4_, and other air pollutants were released with anaerobic decomposition of waste in the MSW landfills. While the concentration of these pollutants have been published to be lower than regulatory limits, any exposure to land fill gases (LFG) such as those of H_2_S and NH_4_, even at lower concentrations, had a negative impact on the respiratory system and the general immunity of children living near the landfill. Children living closer to the landfills showed lower levels of lysozyme associated with exposure to CH_4_ and H_2_S and lower SIgA levels associated with H_2_S and NH_3_. These two factors are measured as they are among the first line of defense in the human body, and their lower levels in children reduced their immunity. They, also, established that as the distance from landfill increases, the effects are reduced (Xu et al. 2018). This experiment yet again establishes the health impact landfills have on young children as a manifestation of a pathology and as an impact on their immune system and its development.

Triassi et al. (2015) conducted a study on the environmental pollution from illegal waste disposal and health effects. Improper landfill management and shipments of illegal waste can have adverse environmental and public health effects. Different handling and disposal operations may result in negative effects arising in land, water, and air pollution. Insufficiently disposed or untreated waste can trigger severe health issues for communities surrounding the disposal zone. Waste leakages can contaminate soils and streams of water and cause air pollution by, i.e., emissions of PTEs and POPs, thereby creating eventually health risks. Other nuisances created by uncontrolled or mismanaged landfills that can negatively impact individuals include local-level effects such as deterioration of the landscape, local water, air pollution, and littering. Therefore, proper and environmentally sound management of landfill is essential for health purposes (Triassi et al. 2015).

A study conducted in Serbia revealed similar findings of high concentration of PTEs, such as Cu and Pb in groundwater and Hg in soil due to the leaching from uncontrolled local MSW landfills. Hg was reported to have high ecological risk for that region (Krčmar et al. 2018).

Melnyk et al. (2014) conducted a study on chemical pollution and toxicity of water samples from stream receiving leachate from a controlled MSW landfill. A relevant factor concerning health effects of landfill management is how much and which population is involved in such risks. Unlike in the case of urban air pollution, exposure to pollution from landfill mismanagement facilities does not affect all the inhabitants of an urban area but only a small proportion of the population residing nearby the landfill. Living in the vicinity of a landfill can pose a health danger to citizens as they may be subjected to pollutants through various routes: inhalation of SoC emitted by the site and contact with water or polluted soil, either directly or through the consumption of products or contaminated water. The greatest issues are illegal, uncontrolled landfills that receive waste at source without any choice (Melnyk et al. 2014).

Palmiotto et al. (2014) conducted a study on the influence of a MSW landfill in the surrounding environment. Landfill has been regarded as the oldest form of waste treatment and the most prevalent technique of structured waste disposal and has remained so in many parts of the globe. A modern landfill is an engineered establishment, specially built and equipped with protected cells. Despite the reality that growing quantities of waste are being reused, recycled, or energetically valued, landfills still play a significant role in the waste management infrastructure of many countries. The degradation of waste in the landfill results in the production of leachate and gases. These emissions pose potential threats to human health and environmental quality. Landfilling has environmental impacts, primarily because of the long-term manufacturing of CH_4_ and leachate (Palmiotto et al. 2014).

A research by Abd El-Salam and Abu-Zuid (2015) on the effect of waste leachate on soil quality in Egypt proposed the need to adjust variables to enhance anaerobic biodegradation leading to leachate stability in relation to ongoing groundwater surveillance and leachate therapy procedures. Landfill construction and management have ecological impacts that can lead to modifications in the landscape, habitat loss, and wildlife displacement. Socio-economic effects of landfills include hazards to public health arising from leachate contamination of the ground or groundwater, the spread of litter into the wider setting, and insufficient recycling operations on site. Nuisances like flies, odors, smoke, and noise are often cited among the reasons why people do not want to live near landfills. However, depending on the real distance from the landfill, landfills are likely to have an adverse impact on housing values (Abd El-Salam and Abu-Zuid 2015).

Furthermore, Rezapour et al. (2018) found that uncontrolled leak of leachate from landfills drastically increased the concentration of various PTEs in the soil which interacted with the crops grown there. They reported that a number of metals were found in moderate quantities, except Cd which was above limits and posed moderate intensity non-carcinogenic risk to the people consuming the wheat. This study however reported that the cancer risk to the local resident was low. This study illustrates the extent of landfilling-generated pollution. The PTEs could interact with the soil system and enter the food chain, thus causing harmful effects to the human population (Rezapour et al. 2018).

Giusti (2009) stated that the ways of exposure that result in health effects associated with waste landfilling are inhalation, consumption, and the food chain. He, also, noted that the health risks associated with individuals directly involved in the waste management system is much higher due to their proximity to the hazard and that the cases of adverse effects are higher among workers than the residents near the landfill. Moreover, he underpinned the fact that the waste management industry has the highest occupational accidents than other professions. For populations living in close proximity to landfills, the risk of birth defects and cancer increased (Giusti 2009).

A study conducted in the island of Mauritius, dealt with the impact of non-hazardous solid waste coming from the only landfill of the island. It was found that vomiting and nausea were consistent symptoms among the population. A large difference in the body mass index of men as compared to their control group was, also, noticed, a pattern that was not observed among women or children, thereby indicating that the effects of pollution can vary on the gender of the individual. Interestingly, it was also found that many other symptoms of health issues were reported; however, they were attributed to either the confounding factors or to a “pan symptom” effect, personal bias. Although this exclusion may be due to the nature of this study being dependent on patient’s information, it provides new dimension to think about personal bias or the placebo effects especially when counteracting seemingly non-threatening diseases associated with landfills, unless proved otherwise by medicinal science (Goorah et al. 2009).

Other studies conducted by various researchers showed that there was an increased risk of malformation of babies among women who lived close to hazardous landfill sites in Washington state and the risk increased among those living in urban areas compared to rural areas (Kuehn et al. 2007).

In the research of Damstra (2002), it was stated that exposure to endocrine-disrupting compounds (EDCs) can put women at risk for breast cancer among other factors, although there are no studies that show a direct increase in the levels of breast cancer with exposure to EDC. However, Damstra claimed that the time of exposure of these chemicals in these women’s lifespan determines the risk. He also reported that studies have shown that exposure to polychlorinated biphenyls (PCBs) in newborn and young children has resulted in neurobehavioral changes, such as immaturity in motor functions, abnormal reflexes, and low psychomotor scores, and these changes may continue into their childhood. He, also, reported that studies suggest that when mothers exposed to low levels of PCBs give birth, the babies have subtle neurobehavioral alterations (Damstra 2002).

Martí (2014) performed a human health risk assessment of a landfill based on volatile organic compounds emission, emission, and soil gas concentration measurements. Direct dumping of untreated waste in rivers, seas, and lakes can cause severe health hazards to accumulate toxic substances in the food chain through the plants and animals that feed on it. Human health may be affected by exposure to hazardous waste, with kids being more susceptible to these pollutants. Indeed, immediate exposure can lead to illnesses through chemical exposure, as chemical waste release into the atmosphere leads to chemical poisoning (Martí 2014).

Agricultural and industrial waste can also pose severe health hazards. Other than this, the co-disposal of municipal, industrial, and hazardous waste can expose individuals to chemical and radioactive risks. Uncollected solid waste can also obstruct the runoff of storm water, leading to the formation of stagnant water bodies that become the disease’s breeding ground. Waste dumped near a source of water also causes water body or groundwater source contamination (Krčmar et al. 2018).

Sharifi et al. (2016) performed a risk assessment on sediment and stream water polluted by toxic metals released by a MSW composting plant. Solid waste disposed of in landfills is generally subjected to complicated biochemical and physical procedures resulting in both leachate and gaseous emissions being produced. When leachate leaves the landfill and reaches water resources, it can lead to pollution of surface water and groundwater. Gas and leachate generation, mainly due to microbial decomposition, climatic circumstances, refuse features, and landfilling activities are unavoidable implications of the practice of solid waste disposal in landfills. In both current and new installations, the migration of gas and leachate away from landfill limits and their release into the atmosphere pose severe environmental concerns. These issues result to fires and explosions, vegetation harm, unpleasant odors, landfill settlement, groundwater pollution, air pollution, and worldwide warming in addition to potential health risks (Sharifi et al. 2016)

Liu et al. (2016) conducted a study on health risk impact analysis of fugitive aromatic compound emissions from the working face of a MSW landfill in China. Over the past three decades, worldwide concern has been growing with regard to the effects of landfill mismanagement on public health. Human exposure to pollution from landfill is thought to be more intense in human life now more than ever. Pollution from landfills can, also, be caused by human activity and natural forces. The significance of environmental factors to the health and well-being of human populations is increasingly apparent. Landfill is a global issue, and it has a huge ability to impact human population health.

Landfill, in the densely settled urban-industrial centers of the more developed countries, reaches its most severe proportions. More than 80% of polluted water was used for irrigation in poor nations around the globe, with only 70–80% of food and living safety in urban and semi-urban-industrial regions (Assou et al. 2014).

Kret et al. (2018) conducted a study on respiratory health survey of a subsurface smoldering landfill. The water we drink is vital to our well-being and a healthy life, but unfortunately polluted water and air are prevalent worldwide. Landfill is tangled with unsustainable anthropogenic activity, leading to significant public health issues. Some of the illnesses connected with landfill pollution are infectious diseases such as cancer, birth defects, and asthma. Environmental health issues are not just a conglomerate of worries about radiological health, treatment of water and wastewater, control of air pollution, disposal of solid waste, and occupational health, but also a danger to future generation (Kret et al. 2018).

By looking at its definition, pollution is considered to be very harmful, too much of which occurs at the incorrect location. However, some erstwhile pollutants are useful in suitable amounts. Aquatic life requires phosphates and other plant nutrients; however, too much of these nutrients and the outcomes of eutrophication are harmful. CO_2_ in the atmosphere helps to maintain the earth warm enough to be habitable, but the accumulation of vast amounts of surplus CO_2_, generated by the use of fossil fuel and other sources, is now threatening to change the climate of the planet. Other pollutants, such as dioxin and PCBs, are so toxic that even the smallest quantities pose health risks, such as cancer and impairment of reproduction. Pollutant releases to the environment are most frequently the casual by-product of some helpful activity, such as electricity generation or cow rearing. This sort of pollution is a form of waste disposal. It happens when the financial expenses of eliminating pollution are greater than the financial advantages, at least the polluter benefits (Zhang et al. 2016).

Although nutrients such as nitrogen and phosphorus are vital to the aquatic habitat, they may trigger over fertilization and accelerate the lakes’ natural aging (eutrophication) cycle. In turn, this acceleration generates an overgrowth of aquatic vegetation, huge overall shifts, and a general change in the biological community from low productivity with many varied species to elevated productivity with big numbers of a few less desirable species (Koda et al. 2017). Bacterial action oxidizes organic carbon that is biodegradable and consumes dissolved oxygen in water which may cause a threat to the aquatic life. In extreme cases where the loading of organic carbon is high, oxygen consumption may result in an oxygen depression that is adequate to cause fish killing and severely interrupt the development of related organisms that require oxygen to survive. A result of this pollution is water hyacinth and other floating aquatic vegetation.

It was deemed appropriate and necessary to tabulate the rest of the articles reviewed in an effort to include as much information as possible on the environmental and health effects associated with landfilling. Table 1 summarizes and depicts a consolidated view of these articles reviewed, together with any associated environmental and/or health impact of the various types of landfills reported therein.

**Table 1:** Environmental and health impacts of landfilling

Article No.	Type of landfill (if provided)	Environmental impact	Health impact	References
1	Non-hazardous waste landfills	-	No suggested excess risk to the residents	Schlosser et al. (2016)
2	Landfill	-	Impaired hepatic health in those with occupational or environmental exposure	Ogunlaja et al. (2019)
			Potential emergence of infectious diseases	
3	MSW landfill	Leachates polluted the soil and surface water but did not reach the groundwater	Odor caused stress, bad mood, annoyance, and a feeling of helplessness to the people living in the vicinity of the landfill	Sánchez-Arias et al. (2019)
		Dust resuspension during waste separation, compaction, and coverage practices of the landfill caused the release of PM_10_ particles causing air pollution	Diseases such as asthma, flu, cough, stomach ache, and skin infections were related to the landfill	
4	Landfill	-	Exposure to two major waste management facilities (landfill and plastic recycling) studied. Proximity to landfill lowered neurodevelopmental scores in children and was associated to toxic metal exposure; increased risk of cancer later in life	Sarigiannis (2017)
5	Regulated and unregulated dumpsites	Leachates with high levels of nitrates, phosphates, PTEs, Mn, Cr, Ni, Cd, and organic compounds which exceeded the US EPA standard for drinking water	Cellular organelles and DNA damages in in vitro cytotoxicity assays in human derived cells	Khalil et al. (2018)
			Upregulation of liver activity enzymes coupled with significant damage expression in the liver, spleen, and bone marrow DNA in mice	
			Molecular damages can cause cancer	
6	Landfill	-	The leachates were found to cause DNA damage, cell death, change in morphology, and detachment from the substratum and cytoplasmic vacuolations in the treated cells	Alimba et al. (2016)
7	Landfill	BPA contamination was found to be the highest near the BPA manufacturing areas and leached into water bodies	BPA was reviewed to cause a number of health issues such as causing diabetes, cardiovascular disease, increased cancer risk, and DNA damage	Huang et al. (2012)
8	Uncontrolled municipal landfill	Leachate containing As, Al, Pb, Cl, NH_4_^+^, Fe, and Mn contaminated underground water, and contamination decreased with increasing distance, and groundwater at a depth of 30 m was not suitable for drinking	-	Han et al. (2014)
9	Landfills	-	Review concluded that the results from landfill studies showed congenital malformations were the most conclusive reports on human health	Giusti (2009)
10	Hazardous waste landfills	Over a long period of time, leachate rate was much higher than short-term leaching	Some metals like Zn, Mn, and Ni had non-carcinogenic effects	Xu et al. (2018)
		Contaminated drinking water	While Pb had both carcinogenic and non-carcinogenic effects	
			The toxicity of the substances varied based on concentration and morphology	
11	MSW landfill	-	PCDDs and PCDFs levels in air were low and did not have any carcinogenic or non-carcinogenic risks in the area surrounding the landfill	Li et al. (2017)
12	MSW landfill	Landfills are the sources of MPs and not a sink for plastics as the MPs were resultants of plastic fragmentation	-	He et al. (2019)
13	MSW landfill	VOCs are also responsible for the formation of tropospheric ozone and SOA (secondary organic aerosols) that causes air quality degradation, odor nuisances in the surrounding areas of landfills, and related psychological stress on inhabitants	Certain VOCs have potential to cause cancer in high concentrations. Studies on impacts of low concentration of VOCs are not conclusive or abundant	Nair et al. (2019)
14	Open “landfill”	Concentrations of PTEs such as Fe, Mn, Cd, and Pb were above allowed limits	-	Alam et al. (2019)
		Soil concentration of Pb, Fe, and Mn were higher, accumulation of Mn and Zn in plants were observed indication bioaccumulation and water had significant levels of all the metals mentioned except for Fe and Pb		
15	Open dumps or “controlled” dumps	Leachate has polluted drinking water wells and underground tanks in the vicinity of San Gaspar site; high biological contamination in leachate from Los Laureles site which crosses an irrigation source; high Pb levels in El Taray site	-	Bernache (2003)
16	MSW landfill	H_2_S was the major contributor to olfactory pollution	The individual carcinogenic and non-carcinogenic effects (sulfur compounds) were lower than permissible limits; however, the combined risk of both was far beyond permissible limits	Wu et al. (2018)
17	Solid waste landfill	-	There are no significant harmful impacts on the population based on the risk assessment model that indicates that the HI for carcinogenic and non-carcinogenic pollutants in the below thresholds	Davoli et al. (2010)
18	Waste dumpsite	-	PBDE poses no to low risk on the population but can cause cancer risks in the future due to their bioaccumulation properties. PCBs showed low-moderate and high potential carcinogenicity depending on the mode of transfer	Hafeez et al. (2016)
19	Open landfill	-	Health risk assessment showed that pathogenic bio-aerosols deposited in adults, while their finer PM affected children. Complaints included cough, chest pain, asthma, aspergillosis etc.	Madhwal et al. (2019)
20	Uncontrolled dumping	Contamination of water canal with Cd, As, Hg, phthalates, bisphenol A, and PAHs above maximum allowed limits from pyrogenic and petrogenic sources	-	Borjac et al. (2019)
21	Open dumping	The geotechnical properties of the soil (maximum dry density, specific gravity, cohesion, CBR, permeability) were significantly deteriorated due to dumping	-	Sharma et al. (2018)
22	Open waste dumping	Alteration of soil properties such as high pH, TDS, and EC. Increase in toxic metal concentration in the soil (Pb, Cu, Ni, Cr, Zn). Plant diversity in the region decreased due to the change in soil characteristics.	-	Ali et al. (2014)
23	MSW dumpsite	Contamination of drinking water with moderately high levels of toxic metal due to percolation of leachate	-	Biswas et al. (2010)
24	Open dumpsite	-	Bio-aerosols containing *Aspergillus fumigatus* and fungi caused chronic cough, chronic phlegm in waste workers with higher prevalence among smokers than non-smokers. It also varied with the waste activity performed	Akpeimeh et al. (2019)
25	Waste dumping	Waste entered water systems such as river	-	Kang et al. (2020)
26	Illegal dumping	-	Increased cancer mortality and congenital malformations were found to be in excess in studies	Marfe and Di Stefano (2016)
27	MSW dumping and burning	Releases CO_2_, CH_4_, SO_2_, NO_X_, CO, NH_3_ in tons and are important air pollutants that causes changes in the climate	Reviewed studies show health impacts such as respiratory disease, heart diseases, and allergic hypersensitivity	Das et al. (2018)
28	Lined landfills	Concentrations of perfluoroalkyl substances were found to be higher in leachate which is of concern as they are persistent	-	Harrad et al. (2019)
29	Landfills	-	Landfills can be a source of dioxin pollution which can cause craniofacial defects. It also has teratogenic effects on exposed populations	(Leśków et al. 2019)
30	Municipal landfill	Landfills are capable of causing air pollution including the release of various metals and hazardous compounds that could be detected with the help of lichens and could have been unnoticed in surveys	-	(Sujetovienė et al. 2019)
31	MSW landfill	Improper drainage systems of landfills could cause migration of the leachate to the underground water	-	Przydatek and Kanownik (2019)
32	MSW landfill	Air pollution	Landfills act as a source of emission of bacterial cells and their endotoxins which can pose a threat to the health and safety of the workers and those living by. The concentrations of these near the landfills varied on a number of factors	Cyprowski et al. (2019)
33	Dumping of wastes and landfilling	Dumping of factory waste consisting of POPs evidently increases its concentration in surrounding air. This is a source of air pollution	-	Navarro et al. (2019)
		If washed down, they could cause water pollution		
34	Dumping of waste	Wastes dumped in the form of landfills after coal mining and processing poses as significant contributors of Hg. They are present in much higher concentration than background levels leading to the pollution of the soils and the land on which it is dumped	-	Antoszczyszyn and Michalska (2016)
35	MSW landfill	Groundwater was contaminated with due to leachate	-	Grygorczuk-Petersons and Wiater (2016)
		This implies that improper lining or absence of results in much groundwater contamination		
36	Landfill	Landfills release micro-pollutants due to the presence of organic compounds in them and their release continues even after their closure posing a risk even after their lifetime	-	Vodyanitskii and Yakovlev (2016)
37	MSW landfill	-	Release of aromatic compounds from MSW landfills increases carcinogenic effects almost to 1.5 km downwind in normal case scenarios and extended up to 4 km downwind in worst case scenarios. This continues to be harmful to populations that can live near these type of landfills in poor countries	Liu et al. (2016)
38	MSW landfill	They contaminated the underground water with hazardous organic pollutants such as PAHs, PCBs, and PCDFs among 82 other contaminant parameters. They also were above legislative limits. This may also shed light to the fact that they are either not maintained or that release of contaminants is hard to control or monitor unless one looks for specific contaminants	-	Indelicato et al. (2017a)
39	Landfill garbage site	Case study of the impact of the leachate on groundwater quality, which was found to be deteriorated	-	Van Giang and Duan (2017)
40	MSW landfill	Groundwater quality was found to be deteriorated in 98.85% of the samples collected near the landfills. This yet again shows the extent of water quality impeder landfills are		Najafi Saleh et al. (2019)
41	Domestic waste landfill	Landfills release greenhouse and toxic gases due to aerobic and anaerobic processes (respiration) under different environmental conditions. This ultimately contributes to the growing problem of global warming	-	Sainova et al. (2019)
42	Illegal dumping and landfilling	Illegal dumping of municipal waste has seen to drastically lower groundwater quality in two out of the five landfill sites observed	-	Naveen and Malik (2019)

## Conclusions

This study aimed at assessing the environmental pollution and health effects associated with waste landfilling. A desk review design was adopted, and information was gathered from the already available sources. The literature review was centered along three themes: waste landfilling, waste landfilling and environmental pollution, and waste landfilling and health issues.

From the reviewed information, it was established that landfills play an important role as far as disposal of solid waste is concerned. It was shown that majority of the countries have adopted landfilling as waste management systems. The literature indicates that some landfills have lining at the bottom to prevent leakage of the waste into the underground water. The present review revealed, also, that landfills are meant to create conducive environment that enhances microorganisms’ activities and thus decomposition of the waste.

Despite the role played by landfills in the waste management sector, the reviewed literature showed that they are linked with environmental pollution. Landfills were seen to have an influence on biodiversity and the flora and fauna, as well as the aquatic life. Literature indicates that landfills are associated with environmental pollutants including mice and other rodents. The gases released from landfills result into air pollution of the area surrounding the establishment, in addition to the release of bio-contaminants. Landfills are, also, associated with pollution of the underground water, especially when the lining at the bottom is not sufficient to prevent leakage of the waste and a large body of literature supports this.

This article investigated, also, the health issues associated with landfilling. It was concluded that through landfills, there are possible chances of emission of gases into the air like CO_2_, H_2_S, CH_4_, and NO_x_. These gases have been associated with respiratory health challenges and some specific types of cancer, e.g., lung cancer. Carcinogenic risks were found to vary between studies but were mostly attributed to the varying characteristics of the landfill. A variety of literature suggests, also, that the environmental pollution caused by landfills creates greater risks to children living in the vicinity of the landfills. Teratogenic effects of certain elements found in the contaminated groundwater were, also, observed. Unarguably, humans produce a large amount of waste, and landfills provide the easiest and relatively efficient way of tackling these waste. However, landfilling has larger deleterious effects that seem to overweigh the benefits it provides. Better technological involvement in waste segregation and appropriate waste management techniques, stronger enforcement of regulations surrounding landfills, and setting up a larger concrete minimum distance for settlements are some of the necessary measures to be seriously considered and taken in the near future.

## Data Availability

Not applicable.

## Nomenclature

CBR: California bearing ratio
EC: Electrical conductivity
EDC: Endocrine-disrupting compounds
GHG: Greenhouse gases
ISQG: Interim sediment quality guidelines
LFG: Landfill gas
LWPI: Landfill water pollution index
MPs: Microplastics
MSW: Municipal solid waste
NCDs: Non-communicable diseases
PBDEs: Polybrominated diphenyl ethers
PCBs: Polychlorinated biphenyls
PCDFs: Polychlorinated dibenzofurans
POPs: Persistent organic pollutants
PTEs: Potentially toxic elements
SoC: Substances of concern
TDS: Total dissolved solids
UNEP: United Nations Environment Programme
US EPA: US Environmental Protection Agency
USA: United States of America
VOCs: Volatile organic compounds
WHO: World Health Organization
Al: Aluminum
As: Arsenic
BPA: Bisphenol A
Cd: Cadmium
CH4: Methane
Cl: Chlorine
CO: Carbon monoxide
Co: Cobalt
Cr: Chromium
Cu: Copper
Fe: Iron
H2S: Hydrogen sulfide
Hg: Mercury
Mn: Manganese
NH3: Ammonia
NH4: Ammonium
Ni: Nickel
NOx: Nitrogen oxides
Pb: Lead
SigA: Secretory immunoglobulin A
SO2: Sulfur dioxide
SOAI: Secondary organic aerosols
Zn: Zinc
